# Editorial: Cognitive Impairment: Therapy Momentum in the Continuum of Life

**DOI:** 10.3389/fphar.2020.618344

**Published:** 2021-01-29

**Authors:** Artemissia-Phoebe Nifli, Magda Tsolaki, Jos Tournoy, Kazuki Ide

**Affiliations:** ^1^ Department of Agriculture Crop Production and Rural Environment, University of Thessaly, Volos, Greece; ^2^ Department of Animal Sciences, University of Thessaly, Larissa, Greece; ^3^ 1st Department of Neurology, School of Medicine, Aristotle University of Thessaloniki, Thessaloniki, Greece; ^4^ Division of Gerontology and Geriatrics, Department of Public Health and Primary Care, KU Leuven, Leuven, Belgium; ^5^ Uehiro Research Division for iPS Cell Ethics, Center for iPS Cell Research and Application (CiRA), Kyoto University, Kyoto, Japan; ^6^ The Institute of Natural Sciences, College of Humanities and Sciences, Nihon University, Tokyo, Japan; ^7^ Department of Drug Evaluation and Informatics, Graduate School of Pharmaceutical Sciences, University of Shizuoka, Shizuoka, Japan

**Keywords:** dementia, cognitive impairment, organoid, epileptiform, cholinesterase inhibitors, benzodiazepines, adverse drug effects, gene polymorphism

Cognitive impairment is hastily affecting an increasing number of people all over the globe. Despite age being a well-established risk factor, contributing to an aggregate prevalence over time, cognitive impairment is not a direct consequence of old age. Other factors and conditions may lead to mild cognitive impairment (MCI) or trigger the onset of dementia, while the same and additional factors may emerge in the continuum of the disease (Alzheimer’s Association, 2020). Therefore, the goal of this Research Topic is to highlight recent advances in the field of Neuropharmacology, Pharmacoepidemiology and Geriatrics, in order to efficiently prevent or delay the onset of cognitive impairment and improve dementia management in clinical practice.

The issue comprises five contributions from four different countries. In the midst of the coronavirus disease (COVID-19) pandemic, that has emphasized the needs and difficulties in comorbidities management, most of the studies are representative of the abiding problems in the field of cognitive impairment (Wang et al., 2020). The authors explore alternative pharmacological regimens in case of MCI and dementia, propose genetic and physiological markers to monitor the efficiency of the schemes, and employ stochastic models to address biological diversity. They also propose the development of comprehensive experimental models to overcome the limitations of the previous ones.

So far, numerous rodent (mice) models have been developed to study dementia continuum, especially the most common type, Alzheimer’s disease (AD) (King, 2018). These models have been useful in understanding the aggregation of amyloid peptides in plaques and in elucidating partially molecular interplay. Nevertheless, they don’t necessary resonate the course of AD in humans or the formation of tangles. Papaspyropoulos et al. discuss the potential of human pluripotent stem cell-derived organoids to substitute animal models of neurodegeneration and serve as drug screening platforms.

Pharmacoepidemiology studies may be further useful in monitoring the clinical efficacy of active agents in a large scale and provide us with new insights. Cholinesterase inhibitors do not perform well in clinical practice and a high number of patients seem not to respond to the approved therapeutic doses. Polymorphisms, such as that of CYP2D6, may mediate drug metabolism and lead to low drug circulating levels in about two thirds of the cases (Ortner et al., 2020). In this issue, Lu et al. discuss a large array of genetic polymorphisms, such as CYPs and ATP-binding cassette transporters, as well as those involved in acetyl-or butyrylcholinesterase constitutive signaling and inhibition that influence donepezil PK/PD. They also review sex and race differences and comment on the lack of longitudinal and combined evidence. In line with the latter, Zhang et al. present a meta-analysis exploring AD therapy outcomes, taking into account drug efficacy, tolerance and patient compliance. By employing Markovian chains, correcting for the aforementioned inter-individual variability, they show that each monotherapy or combined treatment in mild to moderate AD presents discrete benefits, and that both the rarity of adverse effects, as in the case of EGb761, and efficacy may guide patient compliance.

The additional management of neuropsychiatric manifestations in people living with dementia is complex. As Zhang et al. conclude, the approved dementia therapies have no significant impact on the array. Natural extracts, such as of *Ginkgo biloba* leaves (EGb761) or *Crocus sativus* L. stigmata (Tsolaki et al., 2016) are promising, but large-scale randomized controlled trials are needed. The use of benzodiazepines, although effective in short-term, has been linked to cognitive decline, and special considerations should be made accordingly in those suffering from anxiety disorders, depression, or sleep disturbances (Salzman, 2020). Federico et al. confirm a substantial impairment of verbal memory in middle-age adult benzodiazepine abusers, and the subsequent improvement of multiple memory domains at the end of one-week clonazepam-flumazenil regimen. It is not clear though whether and at which stage flumazenil could be used to improve memory impairment in dementia patients considering the extensive GABA_A_ergic remodeling (Li et al., 2016). Occasionally, compensatory inhibitory mechanisms may also develop in a subgroup of individuals that experience seizures. Horvath et al. discuss further patterns of interictal or subclinical epileptiform activity, detected in many cognitive disorders, and their primary or secondary role in memory formation through disruption of sleep-memory consolidation. Such information may shape future studies exploring the course of neurodegeneration, shed light on aspects of behavioral and psychiatric dementia symptoms and potentially revolutionize the field of antiepileptic therapy.

In summary, the current Research Topic features the challenges that may appear in view or due to cognitive impairment and the need for comprehensive monitoring and management in the continuum of life, especially when circumstantial pharmacotherapy may be of long-term consequences and advantages.

## Author Contributions

All authors listed have made a substantial, direct, and intellectual contribution to the work and approved it for publication.

## Conflict of Interest

The authors declare that the research was conducted in the absence of any commercial or financial relationships that could be construed as a potential conflict of interest.
